# Suboptimal MMR2 vaccine coverage in six counties in Norway detected through the national immunisation registry, April 2014 to April 2017

**DOI:** 10.2807/1560-7917.ES.2017.22.17.30518

**Published:** 2017-04-27

**Authors:** Maria Hagerup-Jenssen, Sigrun Kongsrud, Øystein Rolandsen Riise

**Affiliations:** 1 Department of Infectious Disease Registries, Norwegian Institute of Public Health, Oslo, Noway; 2 Department of Vaccine Preventable Diseases, Norwegian Institute of Public Health, Oslo, Noway

**Keywords:** Immunisation registry, Vaccination Coverage, SYSVAK, Rule engine, measles-mumps-rubella (MMR) vaccine, RVC

## Abstract

In 2014, Norway became aware of potential low vaccination coverage for the second dose of measles-mumps-rubella vaccine (MMR2) in six of 19 counties. This was detected by comparing the national coverage (NC) for 16-year-olds extracted from the national immunisation registry SYSVAK with the annual status update for elimination of measles and rubella (ASU) reported to the World Health Organization (WHO). The existing method for calculating NC in 2014 did not show MMR2 coverage. ASU reporting on MMR2 was significantly lower then the NC and below the WHO-recommended 95% coverage. SYSVAK is based on the Norwegian personal identification numbers, which allows monitoring of vaccinations at aggregateded as well as individual level. It is an important tool for active surveillance of the performance of the Norwegian Childhood Immunisation Programme (NCIP). The method for calculating NC was improved in 2015 to reflect MMR2 coverage for 16-year-olds. As a result, Norway has improved its real-time surveillance and monitoring of the actual MMR2 coverage also through SYSVAK (the annual publication of NC). Vaccinators receive feedback for follow-up if 15-year-olds are missing MMR2. In 2017, only three counties had an MMR2 coverage below 90%.

## Background

### The Norwegian national immunisation registry

The Norwegian immunisation registry SYSVAK is a national Immunisation Information System (IIS) administered by the Norwegian Institute of Public Health (NIPH) [1]. SYSVAK is legally anchored in the Norwegian law for Health Registries [2] and the SYSVAK regulation [3]. It has been nationwide since 1995 and covers all vaccinations in all age groups. Registrations of vaccinations in SYSVAK are based on the unique personal identification numbers assigned to people registered in the National Registry (population registry of Norway [4]). Since November 2015, SYSVAK has also covered persons applying for asylum in Norway. The population of Norway was 5.2 million people on 1 January 2017 [5].

It is mandatory for health personnel to report all vaccinations offered through the Norwegian Childhood Immunisation Programme (NCIP) [6] to SYSVAK [3]; consent from the vaccinee is not required. On 31 December 2016, SYSVAK contained more than 34 million vaccine entries for more than 4.1 million persons. SYSVAK offers the possibility to produce a snapshot status of the vaccination coverage against a disease at any given time. This can be done for the Norwegian population in general, for targeted geographical areas (at national, county, municipality and district level) and at an individual level. For further details on SYSVAK see Trogstad et al. [1].

### The Norwegian childhood immunisation programme

Measles vaccine was introduced in the NCIP in 1969. Rubella vaccine has been offered to girls since 1978. In 1983, two doses of the measles-mumps-rubella combination vaccine (MMR) was introduced to both sexes and replaced the monovalent vaccines. The current NCIP foresees MMR1 at age 15 months and MMR2 at age 11–12 years. It is primarily public healthcare stations and school healthcare services who offer NCIP vaccinations in Norway. All services, including vaccinations, are voluntary and free of charge.

All countries in the World Health Organization (WHO) European Region have committed to eliminate measles and rubella by 2015. One of the strategies is to achieve and sustain a very high coverage of at least 95% with two doses of measles and at least one dose of rubella vaccine.

### Vaccination coverage in Norway

National coverage (NC) for MMR in Norway is published for ages 2, 9 and 16 years. During the past decade, NC for MMR at age 16 years varied between 91% and 95%. NC for 16 year-olds as reported from SYSVAK before 2015 did not specifically show MMR2 coverage because 16-year-olds would also appear as fully vaccinated if they had only received MMR1 in the past 9 years. However, MMR2 coverage is required in the annual status updates for elimination of measles and rubella (ASU) sent to the WHO Regional Office for Europe (WHO/Europe). In 2015, Norway received feedback from the WHO/Europe Regional Verification Commission for measles and rubella elimination (RVC) that the population immunity was considered alarmingly low in parts of the country, based on ASU reporting (sent in April 2014 for 2013) [7]. The ASU report for 2013 showed low MMR2 vaccination coverage of below 90% in six of 19 counties (range: 87–89%). 

**Figure 1:**
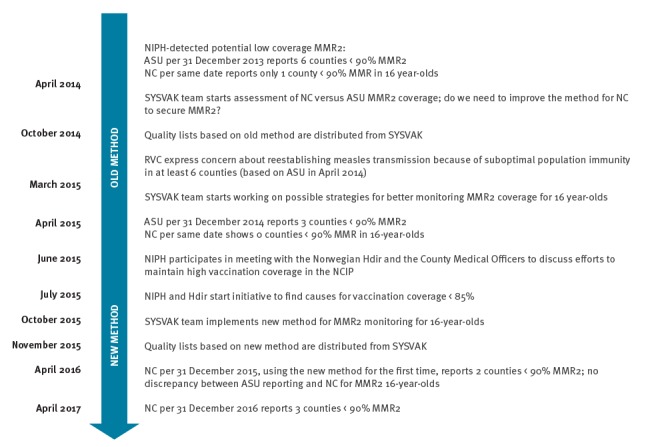
Timeline of corrective actions for MMR2 coverage, Norway, 2014–17

ASU: annual status updates for elimination of measles and rubella; Hdir: Directorate for Health; MMR2: measles-mumps-rubella vaccine second dose; NC: national coverage; NCIP: Norwegian Childhood Immunisation Programme; NIPH: Norwegian Institute of Public Health; RVC: Regional Verification Commission for Measles and Rubella Elimination; SYSVAK: The Norwegian immunisation registry.

We aim here to describe corrective actions taken as a result of the RVC conclusions, in particular changes in the method for assessing MMR2 coverage. Figure 1 shows the main events from the relevant timeperiod 2014–2017.

Quality lists from SYSVAK show children in a cohort not fully vaccinated according to the age and NCIP, or unvaccinated children.

## Method for assessment of vaccination coverage in SYSVAK

### General vaccination coverage assessment

Vaccination coverage in SYSVAK aims to represent actual protection against disease, based on the NCIP. To be considered fully protected, a person needs to have received the vaccine at recommended age and with recommended intervals between doses, according to the NCIP or alternative immunisation schedules (e.g. children who followed a vaccination schedule in another country before residing in Norway).

The coverage is calculated for a given birth cohort and represents the percentage of persons in that cohort who are fully protected against disease. Vaccination coverage can be calculated at any time, for any given cohort and by sex for residents registered in the National Registry in Norway.

#### Foundation: the rule engine

To calculate the real-time vaccination coverage and the extent to which NCIP recommendations are followed, SYSVAK uses a built-in rule engine. Any changes in the NCIP require updates in the rule engine. In order to calculate coverage as accurately as possible, the rule engine takes into account on an individual basis the age at vaccination, intervals between doses and number of doses. It is adjusted to take into account individual deviations from the recommended NCIP, such as age of vaccination and/or type of vaccine.

Vaccines administered within the time range and age limitations accepted in the Summary of Product Characteristics (SmPC) are counted as valid doses in SYSVAK. The rule engine is evaluated on a regular basis and follows the rules below for any of the vaccination schemes in NCIP:

• the minimum age at which the first dose can be counted as a valid dose,
• the minimum age at which a later dose can be counted as a valid dose,
• the minimum interval between a new dose and the previous dose, in order for the new dose to be counted as valid, and
• the period of a vaccination’s validity with respect to the date at which it is administered (i.e. the period of protection offered by the dose).

It is a complex system developed over many years and allows adjustments to improve accuracy in calculating coverage, identify unvaccinated individuals and hence improve vaccination coverage over time.

#### Practical use of the rule engine

The rule engine is a tool that makes the health registry useful on a daily basis as well as in active surveillance during outbreaks. Vaccination coverage can be calculated at any given time using this tool. Coverage can be calculated on a national, regional, municipality or district level using of the National Registry’s information on residency.

National and regional level coverage is published annually for 2-, 9- and 16-year-olds [8] for all NCIP diseases. These data are also reported to WHO through the United Nations Children's Fund (UNICEF)/WHO joint reporting form. Owing to Norwegian data protection regulations, complete coverage data are published only on county level to avoid identifying individuals. Coverage data on municipality and district level for the same age groups are sent to the responsible health personnel in each municipality and district in the major cities in Norway. In addition these data are presented in the Municipal Public Health Statistics Bank [9] in accordance with data protection regulations. 

The rule engine was developed to improve the quality of data in SYSVAK. Quality lists can be produced on municipality and district level. The quality lists identify unvaccinated children as well as children who are not fully vaccinated according to age and NCIP. All registered vaccine doses for the selected diseases are listed. The NIPH produces such quality lists for children aged 2, 8 and 15 years once a year in the autumn. In addition, lists for children aged 15 years are produced annually in the spring. The lists are sent to the responsible health personnel in all municipalities and districts for attention and further follow-up of children resident in their municipality. The quality lists are a tool to monitor and verify the local efforts on immunisation.

The main purpose of the quality lists is to help health personnel ensure that all children are offered the recommended vaccinations according to the NCIP. Furthermore the lists provide quality control of the data reported to SYSVAK and ensure that errors in the registry are rectified. The lists do not give recommendations on further vaccinations. Health personnel may contact counselling services at the NIPH for advice on further immunisation and/or registrations to SYSVAK.

Codes are used to indicate why the persons listed are defined as not fully vaccinated, e.g. minimum age not fulfilled or interval between doses too short. Reported vaccine refusal is also included in the quality lists, but SYSVAK does not have the legal authority to document the reason for refusal.

### Assessing MMR coverage for 16 year-olds

As seen in the timeline in Figure 1, the method for assessing MMR coverage in 16-year-olds was changed in November 2015. Table 1 illustrates how vaccination coverage was calculated in SYSVAK before November 2015. The table includes practical examples of the impact of the method on whether persons appear fully vaccinated or not.

**Table 1:** Method of assessing MMR coverage for 16 year-olds, with examples, Norway, before and after 2015

Description	Pros	Cons	Examples Fully vaccinated at age 16 years	Examples Not fully vaccinated at age 16 years
Old method valid until October 2015				
MMR1: - Minimum age 12 months **- Valid 9 years from date of vaccination** MMR2: - Minimum age 3 years - Valid 20 years from date of vaccination Minimum interval between MMR1 and MMR2: 90 days	Gives snapshot of coverage at any moment. Reflects NCIP^a^ recommendations when MMR1 is given according to NCIP.	For late starters, receiving MMR1 after age 7 years: no alert of missing MMR2. High vaccination coverage does not necessarily mean that MMR2 has been received.	A person who received MMR1 and MMR2 vaccinations according to NCIP. A person with MMR1 at age 7 years (or later) and no MMR2. A person who received MMR1 at age 12 years	A person who received MMR1 at age 6 years. A person who received MMR1 at age 4 years and MMR2 at age 4 years and 1 month.
New method valid from November 2015				
MMR1: - Minimum age 12 months - **Valid until 13 years of age** MMR2: - Minimum age 3 years - Valid 20 years from date of vaccination Minimum interval between MMR1 and MMR2: 90 days	Gives snapshot of coverage at any moment. Secures alignment with NCIP and WHO elimination recommendations for MMR2 (2 doses MMR all children by age 16 years).	New method was quick to implement in the system, but implementation of new practice amongst vaccinators takes time to change. The new method is therefore expected to cause a false decrease in vaccination coverage compared with previous years.	A person who received MMR1 and MMR2 vaccinations according to NCIP. A person who received MMR1 at age 15 months and MMR2 at age 4 years. A person who received MMR1 at age 12 years and MMR2 at age 14 years.	A person with MMR1 at age 7 years (or later) and no MMR2. A person who received MMR1 at age 12 years.

NCIP: Norwegian Childhood Immunisation Programme; WHO: World Health Organization.

^a^ NCIP recommends: MMR1 at age 15 months and MMR2 at age 11–12 years.

### Implementing a new method for assessment of MMR2 coverage

A new method for assessing MMR2 coverage in SYSVAK was implemented in November 2015. The new method also gives a snapshot of coverage at any moment, and secures in addition the previously missing alignment between NCIP and WHO elimination recommendations for MMR2 at the age of 16 years. Table 1 presents the new method and gives practical examples of its impact on individuals.

**Figure 2:**
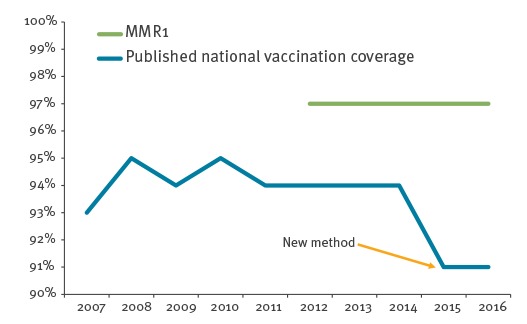
National coverage for MMR and MMR1 coverage for 16-year-olds, Norway, 2007–2016

Figure 2 shows the drop in MMR NC for 16-year-olds following the introduction of the new method (from 94% 2014 to 91% 2015) and the continuously high MMR1 coverage of 97% for the same age group.

Table 2 shows that ASU MMR2 coverage at national level remained unchanged at 91% from 2013 to 2016. However, ASU MMR2 coverage on county level shows a decrease in the number of counties with a coverage below 90% (from six in 2013 to three in 2016). 

**Table 2:** Vaccination coverage before and after introduction of new method, Norway 2013–2016

Year of publication	2014 Coverage for 2013 in %	2015 Coverage for 2014 in %	2016 Coverage for 2015 in %	2017 Coverage for 2016 in %
County	Old method		New method	
Østfold	***87 *** ***(89)***	91 (93)	91	92
Akershus	92 (95)	93 (95)	92	92
Oslo	***89*** (92)	91 (92)	***89***	90
Hedmark	90 (93)	90 (94)	91	90
Oppland	91 (93)	***89*** (91)	91	92
Buskerud	91 (94)	92 (94)	90	90
Vestfold	***88*** (92)	***89*** (93)	90	***88***
Telemark	***87*** (93)	90 (94)	90	***89***
Aust-Agder	***89*** (94)	90 (94)	90	90
Vest-Agder	92 (95)	91 (94)	91	90
Rogaland	94 (95)	94 (95)	94	93
Hordaland	92 (95)	93 (95)	93	93
Sogn og Fjordane	92 (95)	92 (95)	93	90
Møre og Romsdal	92 (94)	93 (95)	93	92
Sør-Trøndelag	93 (94)	93 (95)	94	94
Nord-Trøndelag	91 (94)	91 (95)	91	92
Nordland	91 (94)	91 (94)	90	90
Troms	90 (93)	91 (93)	90	90
Finnmark	***88*** (91)	***84*** (90)	***87***	***88***
**National level**	91 (94)	92 (94)	91	91

ASU: annual status updates for elimination of measles and rubella sent to the World Health Organization; NC: Norwegian national coverage.

The table shows MMR2 coverage reported in the ASU and in parenthesis the reported NC in the same year. Since 2015, ASU and NC coverage have been equal. All vaccination coverage below 90% is marked in italics. Vaccine coverage is calculated per disease. Here, the percentages for measles are shown. Coverage for mumps and rubella can be considered close to equal.

### Additional efforts to improve MMR2 coverage

In addition to the change in method in the IIS, the RVC feedback in 2015 also led to other efforts at the NIPH to secure high MMR2 coverage. The NIPH improved the advice to health professionals on the importance of giving two doses of MMR vaccine to all children. All children 16 years or younger should have two MMR vaccinations, even if the first dose is received later than recommended. Communication efforts were made mainly through the NIPH webpages [10], the Norwegian vaccination guidelines [11], seminars and presentations, as well as different counselling services at the NIPH.

In addition, the NIPH and the Norwegian Directorate of Health initiated an activity mapping local challenges to maintain a high vaccination coverage in June 2015 (Figure 1). A questionnaire was sent to all counties and municipalities to clarify whether the data in SYSVAK showed under-reporting and to highlight the main challenges for improving local vaccination coverage. The questionnaire covered all NCIP vaccines but had a particular focus on MMR2 at age 16 years. 

## Dicussion

We have shown here that SYSVAK detected low MMR2 coverage in six counties (in 2014) and describe actions that were taken to improve this, such as a new method for measuring NC. The new method, which requires a person to have received MMR2 to be considered fully vaccinated at age 16 years, was quick to implement. However, NC published April 2017 still showed low MMR2 coverage in three counties [8].

SYSVAK and its rule engine is unique and based on the NCIP and the vaccine recommendations for Norway. It takes into account other vaccination regimes that have been followed to secure the individual’s vaccination coverage against disease. Before we started ASU reporting on MMR2, we were not aware of the deviation between the NC and MMR2 coverage in the ASU for 16-year-olds. The new method is particularly important in securing MMR2 for individuals that have not had their MMR1 according to the NCIP.

The new method caused a false drop from 2015 to 2016 in published NC because the old method did not show MMR2 coverage for 16-year-olds. It was not a real drop in coverage per se, but it indicated that NC before 2016 had counted 16-year-olds as fully vaccinated even though some had only received MMR1. The drop does not reflect the actual uptake of the MMR vaccination offer in Norway; NC for MMR1 remains high in this age group (97%).

The support for the NCIP is high in the population. Therefore, we believe it may rather be a result of MMR2 not being offered to those who were outside of the regular NCIP MMR regime. The old method was very well adjusted to the NCIP, but not as good for deviations from the NCIP regarding long-term protection against disease secured by two doses of MMR vaccine. The goal of using the new method in combination with other efforts is to ensure that all children receive two doses of MMR. There is currently no system to actively follow up on individual vaccinations after a person has left school and the healthcare services provided by municipality/school, so it is important to catch missing vaccinations before age 16.

So far, we have not seen pockets of unvaccinated children in smaller geographical areas of Norway. By ensuring that the IIS measures vaccination coverage in the best possible way, we should be able to detect such pockets and target them in case of an outbreak where it is important to identify unvaccinated individuals or groups of individuals.

## Future plans and challenges

SYSVAK is considered a complete system offering the basic requirements of an IIS. However, there is potential for further development. Collaboration with expert groups on immunisation registries initiated by the European Centre for Disease Prevention and Control (ECDC) and WHO provides important information on immunisation registries in other countries. The *MesVaccines.net* service in France [12] has functions that could benefit the Norwegian Immunisation registry. Particularly the vaccination recommendations to individuals offered through an online service based on questions and answers are interesting. A similar service on NIPH’s public website, without the need for authentication, could strengthen the current service *My vaccines* [13]. This could have a positive impact on the vaccination coverage of the older population born before the establishment of SYSVAK.

## Conclusion

We have shown how a national IIS could be used to identify and handle low sub-national vaccination coverage. Through a rapid change of the method for assessing MMR2 vaccination coverage, the IIS monitored the measures put in place to improve coverage and thereby contributed to reaching WHO vaccination coverage targets. MMR2 coverage is still below  the 95% WHO target, so efforts to secure two doses of MMR vaccine for all children must continue. Further efforts to increase coverage will be monitored through SYSVAK.
